# Development of genomic markers for monitoring and research on plethodontid salamanders

**DOI:** 10.1371/journal.pone.0336236

**Published:** 2025-11-06

**Authors:** Benjamin M. Fitzpatrick, Kara S. Jones, Aaron W. Aunins, Michael S. Eackles, David C. Kazyak

**Affiliations:** 1 Department of Ecology and Evolutionary Biology, University of Tennessee, Knoxville, Tennessee, United States of America; 2 United States of America Geological Survey, Eastern Ecological Science Center, Kearneysville, West Virginia, United States of America; Texas State University, UNITED STATES OF AMERICA

## Abstract

Despite the importance of plethodontid salamanders and their vulnerability to ongoing environmental change, they are inherently difficult to monitor due to their cryptic nature. Recent advances in genomics have created new opportunities for monitoring of populations and their responses to environmental perturbations. In this study, we developed a new target capture-based genomic panel for the purposes of genetic monitoring in plethodontid salamanders. We demonstrate its utility in several distantly related species and present an example application in two representative species with co-occurring distributions but different ecological attributes and expected patterns of population structure: *Plethodon jordani* and *Desmognathus wrighti*. Although the number of successfully assembled loci declined with phylogenetic distance from the original reference species (*Desmognathus* spp), we obtained high-quality data from thousands of loci from species in all four genera tested (*Desmognathus*, *Plethodon*, *Eurycea*, and *Gyrinophilus*), which span the deepest split in Plethodontidae. Landscape genetic analyses detected weak but statistically significant geographic structure in *P. jordani*, and much stronger geographic structure in *D. wrighti*, as expected based on the lower population density and likely lower dispersal ability of *D. wrighti*. Our target capture panel is broadly applicable across salamanders in Plethodontidae and has the potential to provide data for a wide range of phylogenetic, biogeographic, and population genetics research questions.

## Introduction

Plethodontid salamanders are an important yet cryptic element of many ecosystems. In the Appalachian Mountains, a global hotspot for plethodontid salamander diversity, the biomass of plethodontid salamanders may exceed that of all other vertebrate groups [1–3]. Accordingly, salamanders are key components of regional ecosystems, playing important roles in energy transfer, nutrient cycling, carbon retention, and regulating invertebrate populations [4,5].

As lungless ectotherms, plethodontid salamanders are also particularly sensitive to environmental change. Local climatic and vegetation conditions have strong impacts on the ecology and distribution of populations [e.g., 6,7,8]. Their permeable skin offers minimal protection against unfavorable environmental conditions. Many taxa are found in specialized habitats, with small territories, limited mobility, and low fecundity [9]. As a result, plethodontid salamanders have been proposed as an indicator of environmental integrity [10].

These environmental factors are particularly germane for endemic species restricted to high elevations, including salamander species that are limited to isolated montane habitats [11,12]. For these species, anthropogenic climate change is a hazard, with major range contractions forecast this century. Although uncertainty presents challenges for predicting future occupancy, a range of modeling scenarios consistently predicted a loss of suitable habitat in plethodontid salamanders native to the higher elevations of the southern Appalachian Mountains [13]. Moreover, other threats such as novel pathogens are severely impacting amphibian populations around the globe [14]. These challenges are likely to lead to fragmentation or disappearance of many populations. Population decline and loss of connectivity are leading indicators of extinction risk, thus monitoring changes in abundance, population structure, and genetic diversity may be a valuable component of conservation efforts.

Despite the importance of plethodontid salamanders and their vulnerability to ongoing change, they are inherently difficult to monitor, leaving many aspects of their basic biology poorly known [15]. Plethodontid salamanders in temperate regions are active and accessible at the surface only during part of the year. Even during these periods, most of the population may be underground and not available for sampling. As a result, mortality events are likely to be cryptic and even large demographic changes may go undetected.

Recent advances in genomics have created new opportunities for monitoring populations and their responses to environmental change [16,17]. These approaches can provide information on population structure and gene flow, as well as insight into changes in diversity and effective population size over time [18–20]. Although genomics remains underutilized for population monitoring [21], a growing number of case studies spanning a broad array of taxa highlight the potential utility, including for salamanders [22].

The enormous genomes of salamanders (~10–80 Gb; roughly an order of magnitude larger than the human genome) make it difficult to get adequate depth and coverage to reliably genotype large samples of individuals [23–25]. Reduced representation sequencing approaches such as ddRADseq have shown utility in some salamander groups, particularly for large scale geographic and phylogenetic studies where low coverage data are often adequate [e.g., 26]. However, obtaining high-quality data for some eastern north american plethodontid salamander species has proven to be particularly challenging, likely due to high levels of genetic variation [24].

**Fig 1:**
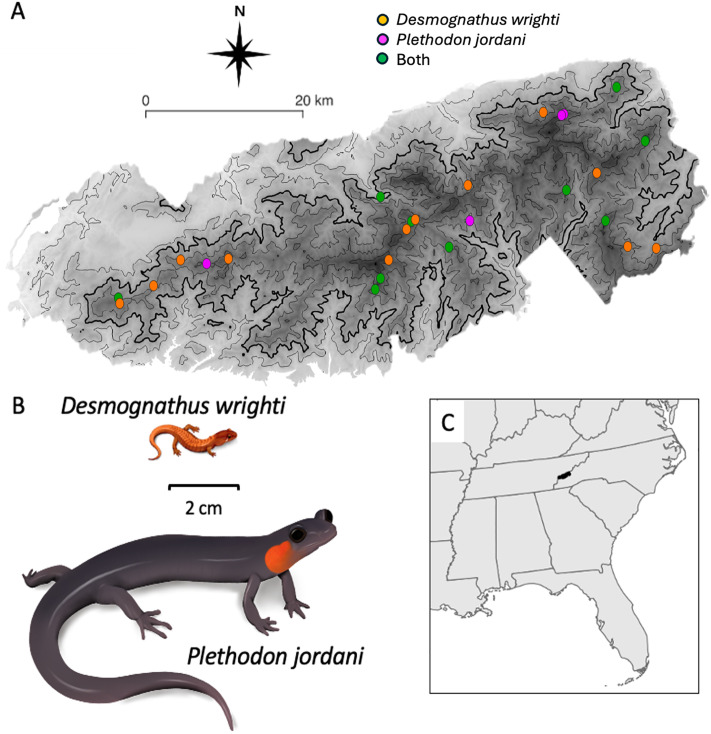
Study area and focal species. **(A)** Sampling sites in Great Smoky Mountains National Park (boundary data from the National Park Service [29]). Contour lines represent 250-m elevation intervals, with the thickest line at 1,000 m to indicate the approximate lower range limit of both species. Darker shading indicates higher elevation. **(B)** Illustrations of study species (used with permission of J. M. Fleming, University of Tennessee, 29 Sep 2025) drawn to relative scale for large adults. **(C)** Location of study area within southeastern North America. All maps are original, created by the authors using the free and open source QGIS (http://www.qgis.org) and R [30,31] with public domain data from the USGS National Map (https://www.usgs.gov/3d-elevation-program/) and the National Park Service [29].

In this study, we developed a new capture-based genomic panel for the purposes of genomic monitoring in plethodontid salamanders. We demonstrate its utility in several distantly related species and present an example application in two representative species with different expected patterns of population structure: *Plethodon jordani* and *Desmognathus wrighti*. Although both taxa are narrowly distributed in the southern Appalachian Mountains and are likely to experience range contractions in the coming decades, there are notable differences in body size (Fig 1) and population density between the species. *Desmognathus wrighti* is a small salamander (adults are 3.7–5.1 cm total length) which typically occurs at low population densities [8,9]. In contrast, *Plethodon jordani* is a relatively large-bodied salamander (adults are 8.5–18.5 cm total length) which is often prolific where it occurs [8,27,28]. Thus, we expect *D. wrighti* to have smaller population size and lower dispersal, which is predicted to result in lower levels of genetic variation and higher levels of geographic structure. We demonstrate that our approach can detect differences in key parameters between these species to provide evidence that the genomic panel can detect ecologically important signals.

## Methods

### Capture bait kit design and initial testing

Putative loci from two ddRAD data sets were used to design the capture bait kit used in this study. The first ddRAD data set included 56 samples from the *Desmognathus quadramaculatus-marmoratus* complex, and 3 *D. carolinensis* from Jones and Weisrock [32], with the addition of 9 *D. folkertsi*, 1 *D. ocoee*, and 1 *D. aeneus* that were sequenced for that study but not previously published. The second ddRAD data set included 8 *D. carolinensis* and 39 *D. fuscus* from Kratovil [33]. Loci from each study were pooled and duplicate loci removed. Full methods for locus generation are available in the original studies cited; in short, both used *EcoRI* and *SphI* restriction enzymes for shearing but different size selection ranges. In both studies, ddRAD sequence data were assembled into putative loci using ipyrad [34] with an 85% similarity clustering threshold. Potential paralogs were removed using standard heterozygosity and depth filters. Hereafter, we use ‘locus’ (or ‘loci’) to refer to the short, continuous DNA sequences inferred from these prior studies to correspond to specific, unique positions in a genome. Loci with BLAST [35] hits to mitochondrial sequences were removed, leaving 10,230 candidate loci between 135 and 290 bp long for use in capture bait design.

Capture baits were designed by Daicel Arbor Biosciences (Ann Arbor, Michigan) using the myBaits “relaxed” requirements (i.e., < 10 BLAST hits for hybridization melting temperature (T_m_) = 62.5–65°C and 4 hits Tm > 65°C), with 80 bp long baits at ~1.8X tiling density. Candidate sequences were masked for repetitive elements and simple repeats using RepeatMasker [36], resulting in a capture kit with 38,313 baits, covering 10,110 loci.

To determine how many homologous, polymorphic loci could be captured with the bait kit, we performed the first test of the kit with DNA from ten individuals collected for a previous study [32], including *D. aeneus*, *D. folkertsi*, *D. monticola*, *D. ocoee*, *D. quadramaculatus* (northern and southern clades *sensu* Jones and Weisrock [32]), *D. wrighti*, *Eurycea guttolineata*, *Gyrinophilus porphyriticus*, and *Plethodon montanus*. DNA was extracted using the DNeasy Blood and Tissue Kit (Qiagen, Hilden, Germany) following the manufacturer’s tissue extraction protocol. DNA quality was visualized on a gel and one sample (PM316; Table S1 in S1 File) with fragmented DNA was included to determine whether capture kit performance was affected by low quality DNA. Libraries were prepared as described below under DNA extraction and sequencing, but the sonication time was adjusted for sample PM316 to account for the DNA already being fragmented. The completed library was sequenced on a NovaSeq 6000 (Illumina, San Diego, CA) with 150 bp paired end reads at a mean depth of 60 million reads per sample to maximize coverage. Reads were mapped to the reference capture loci using BWA-MEM ([37]; as described below under Assembly and variant calling). Although the number of reads per individual was similar across all species, the percentage of reads that were successfully mapped to capture loci (after duplicates, supplementary, and singletons were removed) varied between 15–22% for *Desmognathus* and 4–7% for other genera (S2 Table in S1 File). Loci that did not have any read coverage were removed, leaving 9,756 loci in the final iteration of the capture kit. Original data are available from USGS [38], NCBI BioProject PRJNA1062342, and PRJNA1336299 (https://www.ncbi.nlm.nih.gov/bioproject/).

### Field collections

The second test of the final kit was then applied to 13 samples collected non-lethally (S1 File) from five species in Great Smoky Mountains National Park for this study (Table S1 in S1 File): *D. wrighti* (n = 3), *P. jordani* (n = 4), *P. glutinosus* (n = 2), *P. metcalfi* (n = 2), and *P. teyahalee* (n = 2). DNA was extracted, quality assessed, and library prepped the same as for the first capture kit test. These libraries were sequenced on an Illumina MiSeq 150 bp paired end with a mean depth of ~4.5 million reads per individual. Sequence data are available for these thirteen individuals at NCBI BioProject PRJNA1062342.

For our final datasets, we collected non-lethal tissue samples from *D. wrighti* (*n* = 46, 23 sites), *P. jordani* (*n* = 42, 15 sites), and *P. metcalfi* (n = 4, 2 sites) between June and September 2019 (Fig 1). For sampling purposes, a “site” was defined as a circle with 30m radius, but none of our analyses assume that a sampling site corresponds to a breeding population. Sample sites were broadly dispersed across the known range of *D. wrighti* and *P. jordani* within the Great Smoky Mountains, but loosely clustered into eastern, western, and central groups owing to relative accessibility (Fig 1).

Upon capture, individuals were placed in sterile plastic bags to minimize contact, prevent escape, and reduce the risk of injury to the animal. A small (0.4–1.0 cm) tissue sample from the distal portion of the tail was collected with the clean pinch method ([39] and S1 File) and retained in 95% ethanol. Each individual was handled separately in an unused sterile bag and released alive immediately after tissue collection. All other field gear was cleaned with a 10% bleach solution between sample locations to prevent potential transport of environmental pathogens.

All sampling was conducted in accordance with the SSAR/ASIH guidelines for the use of live amphibians in field research [40] and National Park Service Scientific Research and Collecting Permits GRSM-2019-SCI-2079 and GRSM-2019–2081, National Park Service Institutional Animal Care and Use Committee project approval SER_GRSM_Kazyak_Salamander_2019.A.3, University of Tennessee Institutional Animal Care and Use Committee project 2710, Tennessee Wildlife Resources Agency Scientific Collection Permits 2160 and 2187, and North Carolina Wildlife Resources Commission permit 9709080.

### DNA extraction and sequencing

Genomic DNA was extracted from tail tissue of *P. jordani* and *D. wrighti* collected in the Great Smoky Mountains using the DNEasy blood and tissue kit (Qiagen, Hilden, Germany) and quantified with a Qubit fluorometer using the dsDNA HS assay. For each sample, between 2–3 µg DNA was suspended in 135 µl of 1X TE buffer for mechanical shearing to a target mean fragment distribution of 250 bp with a Covaris E220 Focused Ultrasonicator. Settings for the Covaris were Peak incident power (W) 75, duty factor 10%, cycles per burst 200, and treatment time 180s, using 130 µl of the diluted DNA in one well of a Covaris 96 microTUBE plate. Sheared DNA (200 ng) in a total volume of 50 µl 1X TE was used as input to the NEBNext Ultra II Library prep kit for Illumina following the manufacturer’s instructions. Notable user choices made during the library prep process were: no dilution of adapters for the ligation reaction, size selection and clean-up of the ligation targeting a 200 bp insert size, and between 10–14 cycles for index PCR. Each sample was dual indexed with NEBNext Multiplex unique dual indices and quantified on an Agilent Tapestation 4150 and DNA1000 screentape. After quantitation, the libraries were concentrated to a final volume of 7 µl with a speed-vac.

Hybridization capture and enrichment of the Illumina *P. jordani* and *D. wrighti* libraries was performed using the new capture bait kit (refer to capture bait kit design above) and myBaits User Manual “Standard” protocol (version 5.00; available at arborbiosci.com/mybaits-manual) with some modifications. A hybridization temperature of 65 °C was used for the *D. wrighti* samples, and 63 °C for the *P. jordani* samples. One sample was processed per hybridization reaction, where the volume of baits was diluted 1:8 prior to addition of the 5.5 µl baits. Hybridization on the thermal cycler was extended to 36 hours. “Workflow A” was followed for the enriched library recovery step, where the amount of Buffer E was reduced to 20 µl, and between 12–15 cycles for the Library Amplification step. SPRI bead cleanup of the amplified library used a ratio of 2 parts beads to 1 part PCR product, and an elution volume of 24 µl. The final enriched libraries were combined to a final concentration of 4 µM, and paired end 150 bp sequenced on 1 lane of an Illumina Novaseq 6000. Sequence data for these *P. jordani* and *D. wrighti* samples are available from USGS [38], NCBI BioProject PRJNA1062342, and PRJNA1336299 (https://www.ncbi.nlm.nih.gov/bioproject/).

### Assembly and variant calling

We matched paired end reads and trimmed for adapter contamination with Trimmomatic [41], discarding reads shorter than 40 bases. We then mapped reads to the bait sequences using BWA-MEM [37] and used Picard to identify and remove PCR duplicates [42]. We used bcftools mpileup to call variants [43], excluding those with a quality score below 200 (i.e., the estimated probability that a variant is truly present at the site was at least 1-10^-20^ [43]) or depth below 20 per site. For data analysis, we further restricted the data set to biallelic SNPs (single nucleotide polymorphisms) and discarded individuals with more than 40% missing data. For both species, a small number of individuals were missing more than 60% of the loci (one *D. wrighti* and three *P. jordani*), but all others were missing fewer than 40%. We used this large gap in the distribution to choose our cutoff and did not explore the effects of more restrictive missing data cutoffs for the present analyses. Unfiltered vcf files for both the thirteen test individuals run on the MiSeq, as well as the final set of *D. wrighti* and *P. jordani* are available from USGS [38], NCBI BioProject PRJNA1062342, and PRJNA1336299 (https://www.ncbi.nlm.nih.gov/bioproject/).

### Statistical analyses

To evaluate geographic structure in *D. wrighti* and *P. jordani*, we tested for isolation by distance with Mantel tests [44] and evaluated potential modular population structure using sparse nonnegative matrix factorization (sNMF: [45]), Bayesian ancestry inference with STRUCTURE [46] and multivariate ordination [47]. Following from our sampling protocol, we used individual-based analyses rather than grouping individuals into arbitrary populations. We estimated pairwise individual genetic distances as the mean nucleotide sharing distance across loci, *d*_*mn*_ (called GENPOFAD in [48]). This approach uses all variant sites yet still weights each locus equally. In practice, allele-sharing distances estimated from one SNP per locus were highly correlated with the mean nucleotide sharing distances (S1 File).

We performed Mantel tests of individual *d*_*mn*_ vs. geographic distance (km) using the mantel.randtest function in ade4 [49] with *p*-values estimated from 9999 matrix randomizations. To compare the two species, we estimated 95% confidence intervals for the correlation coefficient using the pivotal method and 1,000 bootstrap replicates [50].

To quantify support for modular population structure with sNMF, we computed the cross-entropy loss function for each of 100 replicate optimizations for each hypothetical number of ancestral populations (*k*) from 1 to 5. Better fitting models have lower cross-entropy, so we saved the replicate with the lowest value to visualize the estimated ancestry proportions [47]. In principle, sNMF fits the same population admixture model as STRUCTURE [46], so for comparison we also used STRUCTURE to estimate admixture proportions for each value of *k*. For both methods we used one randomly sampled SNP per locus. For each species dataset and each value of k from 1 to 5, we ran STRUCTURE for 10 independent replicates of 4 million MCMC steps, discarding the first 1 million as burn-in. We then used STRUCTURE HARVESTER to quantify relative support for each *k* [51].

Finally, we used distance-based ordination (principal coordinates analysis) to visualize multivariate patterns of genetic variation without explicit population genetic assumptions [47]. We used the dbrda function in the vegan package [52] in R [31] with the pairwise nucleotide distance matrix as input with no predictor variables. We compared each analysis to the map of sampling locations to evaluate how well patterns of individual genetic similarity reflect spatial patterns that might merit further research.

## Results

### Bait panel performance

**Fig 2:**
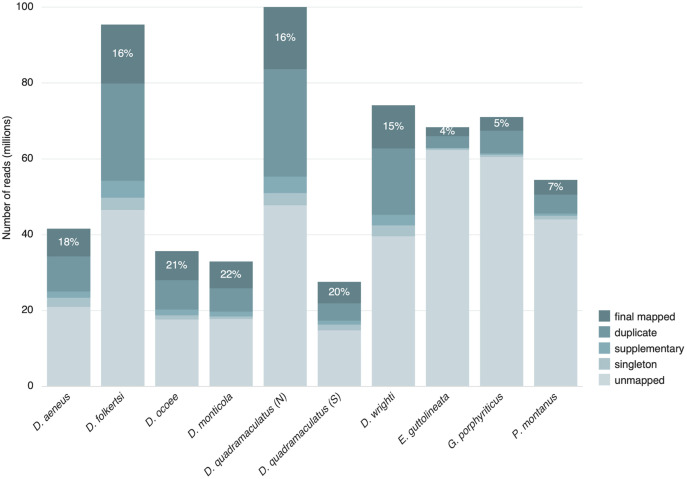
Performance of the preliminary target capture kit across the Plethodontidae. Proportion of reads from first capture run that were kept (final mapped) or discarded because they were optical duplicates, supplementary (mapped to more than one locus), singleton (read missing mate pair), or unmapped. Number on each bar indicates the percentage of reads that were retained after processing (i.e., final mapped). N and S after *D. quadramaculatus* refer to the northern and southern clades, respectively.

Our target capture kit successfully captured on-target reads for every species tested, though the percentage of reads on target was lower outside the *Desmognathus* species used to design the baits (Fig 2). This pattern remained true with the more refined version of the kit (Table 1). Moreover, including *Desmognathus* and *Plethodon* in the same capture batch appears to have biased the number of reads towards *Desmognathus* (Table 1). For our larger dataset, we prepared equal numbers (*n* = 46) of *Plethodon* and *D. wrighti* separately (and with different hybridization temperatures of 65 °C and 63 °C, respectively), and this resulted in a more similar total number of reads for each genus (1.64 x 10^9^ and 1.25 x 10^9^ raw reads, respectively).

**Table 1:** Performance of the final target capture kit for 13 samples sequenced for the second test.

Sample ID	Species	Number of reads	Percent of reads mapped	Final percent mapped *
GSM19044	*P. metcalfi*	1,696,799	11.4%	9.6%
GSM19095	*P. metcalfi*	3,006,724	12.6%	9.4%
GSM19101	*P. teyahalee*	2,651,983	11.3%	9.3%
GSM19113	*P. glutinosus*	3,093,112	12.3%	9.3%
GSM19401	*P. teyahalee*	3,279,227	11.9%	9.5%
GSM19407	*P. glutinosus*	3,125,126	11.8%	9.4%
PJO119	*P. jordani*	2,743,644	11.1%	8.2%
PJO291	*P. jordani*	4,931,164	10.8%	8.1%
PJOIK7	*P. jordani*	4,580,146	11.2%	8.2%
PJOYCG3	*P. jordani*	5,434,220	11.2%	8.2%
DWR12	*D. wrighti*	8,364,299	35.4%	19.0%
DWR163	*D. wrighti*	9,908,731	38.8%	17.9%
DWR63	*D. wrighti*	8,184,084	36.9%	18.2%

* Percent mapped after reads removed that were not properly paired or where mates were mapped to another chromosome.

After mapping and quality control, our final dataset for *D. wrighti* included 7286 loci and 45 individuals. Our final dataset for *P. jordani* included 4402 loci and 39 individuals (four of the original 46 were *P. metcalfi* and therefore excluded for this analysis independently of quality control). These final datasets had low levels of missing data (mean 17.6% of loci per individual for *D. wrighti* and 13.9% for *P. jordani*). The number of SNPs per locus was often large (mean = 12.4 and 8.8 for *D. wrighti* and *P. jordani*, respectively) and significantly different between species (Wilcoxon *W* = 13008554, *p* << 0.0001).

### Comparative analysis of population structure

**Fig 3:**
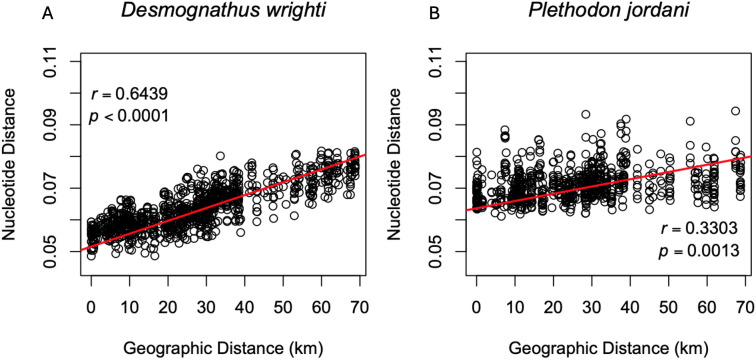
Correlations between individual genetic distance and geographic distance. *P*-values for *Desmognathus wrighti* (left) and *Plethodon jordani* (right) as estimated from Mantel randomization tests with 9999 replicates. Red trend lines were fitted with least squares regression.

Both study species exhibited statistically significant patterns of isolation by distance (Fig 3), however the correlation was much stronger for *D. wrighti* (bootstrap 95% CI: 0.5815–0.7208) than for *P. jordani* (0.2487–0.4025). In addition, the distribution of pairwise nucleotide distances illustrate that the *D. wrighti* within a local area (e.g., within 10 km) were less genetically variable than *P. jordani* within the same range of distances (Fig 3).

**Fig 4:**
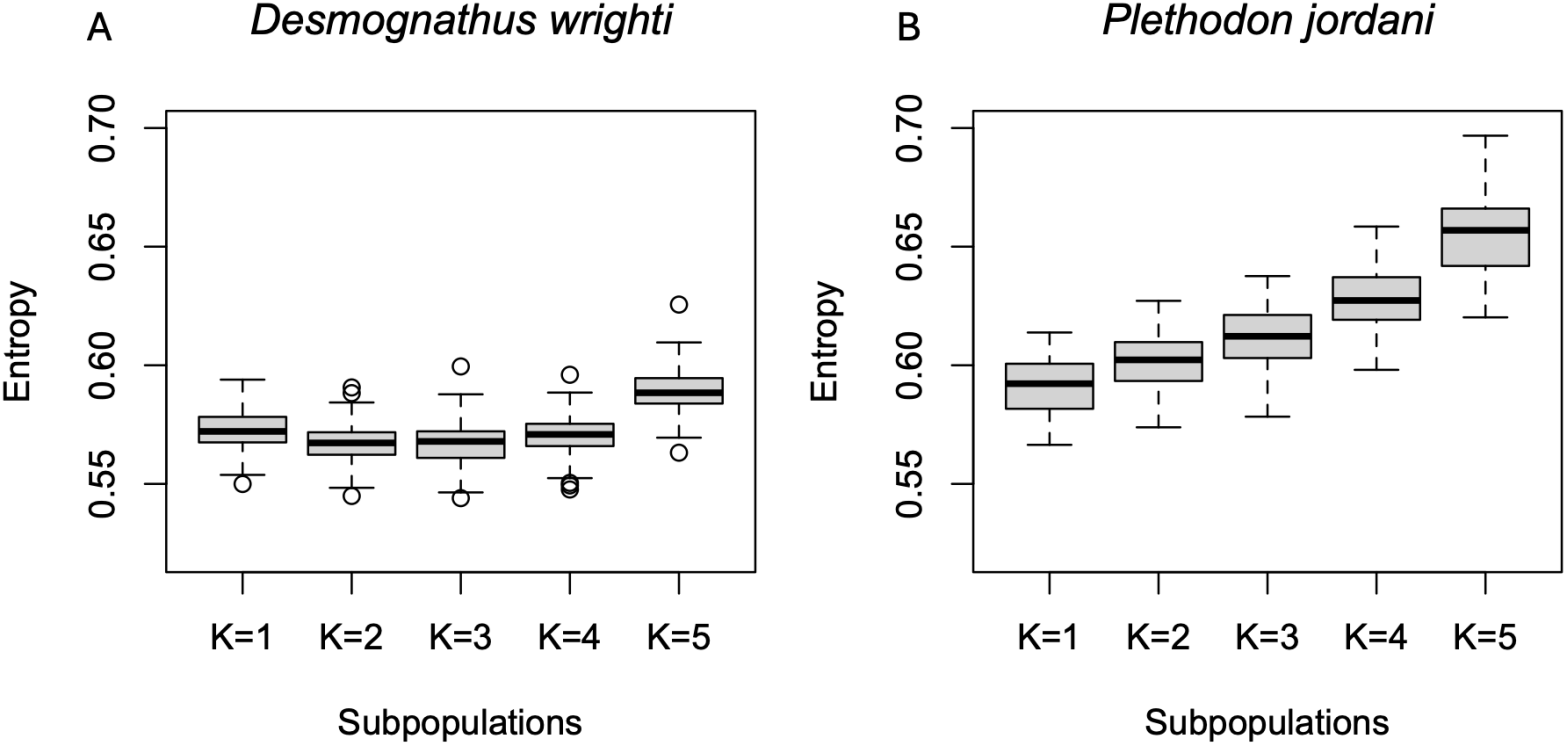
Strength of evidence for different numbers of ancestral populations. Results of cross entropy estimates for each of 100 replicates for different numbers of ancestral populations of *Desmognathus wrighti* (left) and *Plethodon jordani* (right) with the sNMF algorithm [45]. Better fit models have lower entropy.

**Fig 5:**
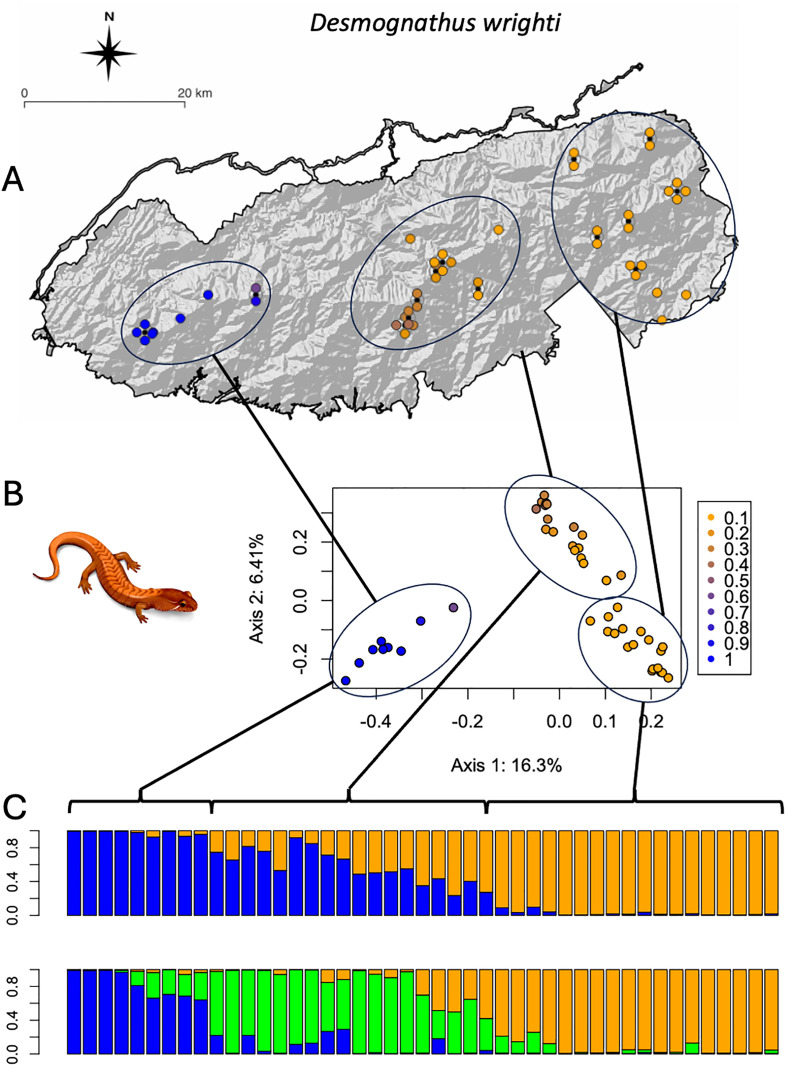
Population structure of *Desmognathus wrighti.* **(A)** Map of sampling locations shaded according to ancestry estimates from the admixture model with k = 2 fitted using sNMF [45]. Locations with more than one individual are shown as black dots with individual shaded circles arranged symmetrically around them. **(B)** PCoA of the pairwise genetic distance matrix, also shaded according to the admixture model with K = 2 fitted via sNMF. **(C)** Barplots of ancestry estimates according to the admixture model in STRUCTURE. Individuals in the barplots are sorted by longitude (west to east) and ellipses connected by lines illustrate corresponding groups of individuals across the graphs. *D. wrighti* illustration used with permission of J. M. Fleming, University of Tennessee, 29 Sep 2025. Original map created by the authors using the free and open source QGIS (http://www.qgis.org) with public domain data from the USGS National Map (https://www.usgs.gov/3d-elevation-program/) and the National Park Service [29].

The best fit number of ancestral subpopulations using the sNMF algorithm supports *k* = 2 or 3 for *D. wrighti*, but no modular structure (*k* = 1) for *P. jordani* (Fig 4). The Bayesian algorithm of STRUCTURE produced similar results for *D. wrighti*, with the delta K method [53] supporting *k* = 2 (S1 File). The first and second multivariate ordination axes largely reflected the model-based ancestry estimates for *D. wrighti*, and both kinds of analysis were consistent with genetic differentiation across the long East-West axis of the Great Smoky Mountains (Fig 5). Ancestry estimates (admixture proportions) suggest a continuum rather than sharply differentiated groups of genotypes, and the appearance of clusters in the ordination plot corresponds to our clustered sampling design, which is an artifact of accessibility rather than the actual distribution of the salamanders (Fig 5).

**Fig 6:**
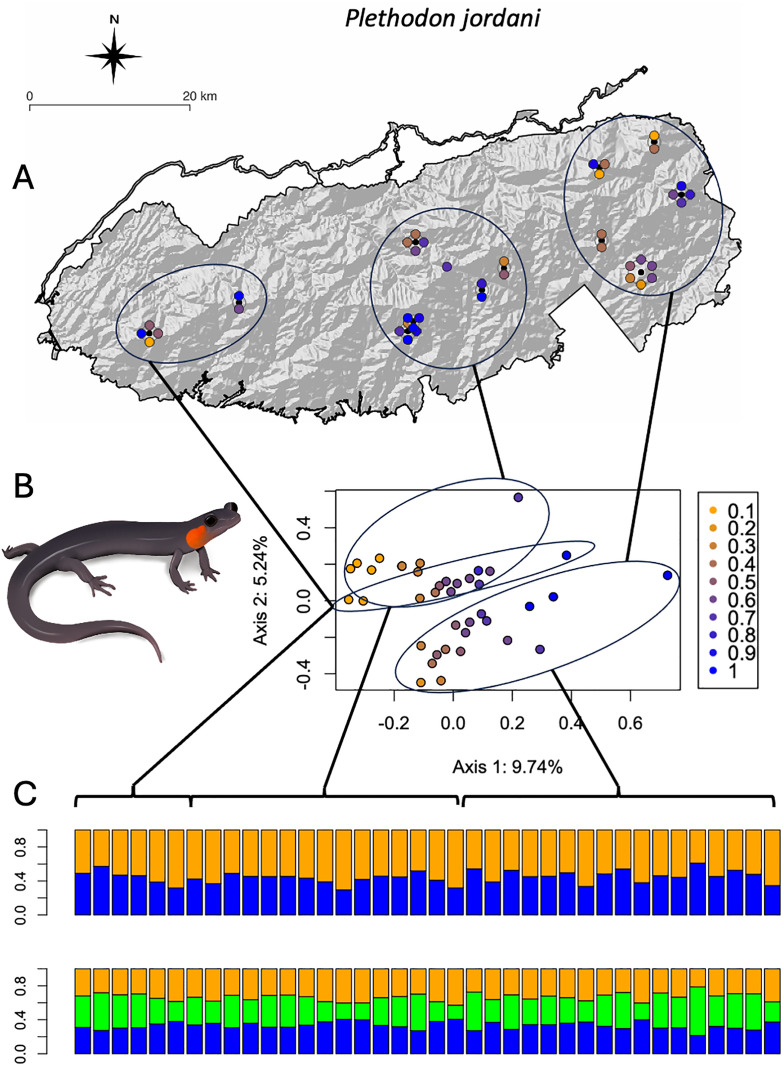
Population structure of *Plethodon jordani.* **(A)** Map of sampling locations shaded according to ancestry estimates from the admixture model with k = 2 fitted via sNMF [45]. Locations with more than one individual are shown as black dots with individual shaded circles arranged symmetrically around them. **(B)** PCoA of the pairwise genetic distance matrix, also shaded according to the admixture model with K = 2 fitted using sNMF. **(C)** Barplots of ancestry estimates according to the admixture model in STRUCTURE. Individuals in the barplots are sorted by longitude (west to east) and ellipses connected by lines illustrate corresponding groups of individuals across the graphs. *P. jordani* illustration used with permission of J. M. Fleming, University of Tennessee, 29 Sep 2025. Original map created by the authors using the free and open source QGIS (http://www.qgis.org) with public domain data from the USGS National Map (https://www.usgs.gov/3d-elevation-program/) and the National Park Service [29].

For *P. jordani,* neither sNMF (Fig 4) nor STRUCTURE (Fig 6, S1 File) indicated any support for *k* > 1. The first two ordination axes explained less of the variation among *P. jordani* than for *D. wrighti* (Figs 4 and 5). As for *D. wrighti*, the first axis for *P. jordani* was associated with the sNMF ancestry estimates for *k* = 2. The second axis suggests weak differentiation between the easternmost third of the Great Smoky Mountains and remainder of the distribution (Fig 6).

## Discussion

Based on our results, the target capture approach with our new bait kit is an effective means to repeatedly sequence an informative set of loci in our focal species. Our final datasets had very low missingness, and read depth was high (over 100x in both species), facilitating population genetic analyses that depend on accurate genotype calls and complete data matrices. Our kit worked for obtaining reads from species across the deepest phylogenetic split in Plethodontidae (i.e., *Eurycea* and *Gyrinophilus* vs. *Desmognathus* and *Plethodon*), corresponding to a divergence time of approximately 74 million years ago (median of 19 studies, credible range 61–86.8 [54]). However, the percentage of reads on target dropped precipitously with evolutionary distance from the *Desmognathus* species used to design the baits (Fig 2). For applications requiring 2,000–4,000 loci, this set of baits is likely to work for most plethodontid salamanders and might be a better starting point than general purpose methods for developing a panel of markers tuned for a particular plethodontid taxon.

Other targeted capture methods have been applied to plethodontid salamanders. Pyron and colleagues used AHE (anchored hybrid enrichment) for phylogenetic analyses of *Desmognathus* [55,56]. AHE is a proprietary protocol, generally targeting under 600 loci, and most often used in phylogenetics rather than population genetics. Newman and Austin [57] used UCE (ultra-conserved element) sequencing for an analysis of phylogeography and species delimitation in *Plethodon serratus*. They resolved under 2,000 loci, which appears typical for the approach [58]. UCEs are also more often used in phylogenetics than population genetics because they are associated with slowly evolving regions of the genome [59] and might not provide a representative sample of the genetic variation in a species [60]. In contrast, target capture based on RADSeq, as we have employed here, has proven to be useful in population genetic analyses across a wide range of taxa [61,62]. In comparison, direct analyses of ddRAD data in plethodontids often yield over 20,000 putative loci, but suffer from high fractions of missing data per individual such that some pairs of samples have few loci in common [24,26,32,63,64].

For two high-elevation terrestrial plethodontids, we show isolation by distance across the continuous distribution of suitable habitat above approximately 1,000 m in the Great Smoky Mountains, but also significant differences between the species (Figs 3–6). Both species are continuously distributed across the same habitat (forest above ~900m) in the Great Smoky Mountains. Our results support the hypothesis that the smaller and less abundant *D. wrighti* has less genetic variation within a local area and greater differentiation between more distant localities (Figs 3–5). In contrast, the relatively large and highly abundant *P. jordani* exhibits greater levels of individual variation and little spatial genetic structure across its entire range (Figs 3,4,6). Many terrestrial plethodontids tend to be sedentary and are thought to have short individual dispersal distances [9]. However, longer individual dispersal events are notoriously difficult to detect [65]. Moreover, the effect of gene flow on population differentiation scales with the number of dispersers (i.e., the product of immigration rate and population size *Nm*), such that a small rate of dispersal can maintain genetic similarity when population densities are large [66]. Thus, the relative spatial homogeneity of *P. jordani* may be explained by contiguous high densities across the higher elevations of the Great Smoky Mountains crest, even if expected dispersal distance is a small fraction of the species range. Tilley [67] showed a similar pattern of relative homogeneity in allozyme data for *Desmognathus imitator,* another high-density species in the Great Smoky Mountains.

There are at least two alternative explanations to consider for the spatial homogeneity of *P. jordani* in our dataset. First, *P. jordani* is known to hybridize with other *Plethodon* (predominantly *P. teyahalee*) at the margins of its range [68,69]. If salamanders all along the periphery are systematically more likely to carry alleles from the lower elevation *P. teyahalee*, then the allele-sharing distances among samples from opposite ends of the geographic range might be reduced as a function of similar introgression patterns rather than as a function of gene flow within *P. jordani*. *Desmognathus wrighti* is not known to hybridize with any other species, but the possibility of introgression might affect the interpretation of landscape genetic patterns in many species.

Second, the subset of target loci resolved for *P. jordani* might be a non-random sample of the genome that is particularly conservative because faster evolving loci may have failed to bind to the baits or assemble to the reference. Although this proposition is supported by the lower average number of SNPs per locus in the *P. jordani* dataset relative to the *D. wrighti* dataset, there is still more individual genetic variation in the *P. jordani* dataset (Fig 3). That is, the loci sequenced in *P. jordani* do not lack variation, the variation is simply not spatially structured to the same extent as seen in *D. wrighti*. In contrast to our results, a STRUCTURE analysis of nine microsatellite loci in *P. jordani* [70] showed support for *k* = 3 and a pattern similar to what we show here for *D. wrighti* (Fig 5). However, those nine markers were chosen non-randomly based on differentiation between two test populations and therefore might overestimate geographic structure [71].

Our analyses illustrate that population and landscape genetics support inferences about plethodontid biology, and our bait panel can facilitate the broad application of multilocus population genetics in plethodontids. The use of target capture sequencing can support more reliable and higher resolution population genetic analyses than non-targeted methods because it focuses sequencing effort on a modest number of specific loci, which is a major advantage for organisms with large and complex genomes [23]. Moreover, target capture does not rely on restriction enzyme sites, which can be polymorphic within and between species of interest, resulting in non-random missing data patterns [e.g., 72, 73]. Our bait panel is a tool that supports increasing knowledge of ecological, evolutionary, and conservation genetics in plethodontids.

With under 10,000 loci, this bait panel cannot offer true genomic monitoring or risk assessment in the sense of scanning for deleterious alleles or other fine grain indicators of organismal fitness [74]. However, with this number of loci, greater read depth per locus facilitates more reliable genotyping and potentially superior resolution of fine scale population structure [25, 75]. Thus, our panel could be used to test for changes in population density and connectivity that might be hard to detect in small secretive animals. Although population genetic patterns have inherent time lags before showing effects of demographic changes [18,76], marker systems similar to ours have been shown to be sensitive to anthropogenic effects on landscapes at small spatial scales in other salamanders [e.g., 77].

The terrestrial plethodontids studied here do not have the metapopulation structure inherent to pond-breeding amphibians and therefore require explicit landscape genetic analyses for continuously distributed populations [78,79]. With finer scale sampling than our example datasets, our markers could be used to estimate genetic neighborhood size and local densities [75, 80], test potential barriers to gene flow that may arise with climate and habitat change, or identify legacy effects of past habitat disturbance on gene flow [77,81]. For the species included in this study, sampling effort across the lowest elevation ridges along the Great Smoky Mountains crest can facilitate comparison of core and marginal habitats. Marginal habitats would include low elevation sites at risk of climate change effects [13], stands of Eastern Hemlock (*Tsuga canadensis*) suffering the effects of invasive pests [8], and areas recovering from catastrophic fire [27].

Currently, many statistical methods use a single SNP per locus to preserve the assumption of Mendelian independence [e.g., 47]. However, with the relatively large number of SNPs per locus evident in our data, methods that can use all of that information will likely provide greater resolution [48]. Our distance-based analyses using *d*_*mn*_ used all variable sites but lacked explicit population genetic parameters. Population genetic models such as STRUCTURE can accommodate linked variants, and many methods are being developed to take advantage of phased haplotype data [e.g., 82].

Experimenting with library preparation approaches could enhance the efficiency, reliability, and cost-effectiveness of target capture. For example, C0t-1 DNA for the group of interest can be isolated and used in place of standard blockers to hybridize with highly repetitive DNA to reduce non-target sequence interference during library preparation [23]. Hybridization time, temperature, and number of samples per reaction can also affect capture efficiency. Preliminary experiments can also be used to identify and remove baits with poor performance, which would tend to increase coverage of remaining loci.

Our capture panel represents a potential tool for genetic monitoring of plethodontid salamanders. Through efficient and repeatable genotyping of thousands of SNP loci, key population metrics can be estimated and tracked through time. Metrics such as effective population size, within-population diversity (e.g., heterozygosity), and among-population diversity (e.g., differentiation) can reveal management-relevant changes in demography and gene flow that may otherwise be cryptic [83]. However, careful consideration of study design is warranted because spatial population structure – such as we documented for *D. wrighti* in this study – can affect inferences derived from any of these metrics. Fixed monitoring sites may alleviate this concern and also allow for the parallel development of demographic time series (e.g., catch-per-unit-effort) during capture surveys to collect DNA samples. Such a monitoring program may be most informative at tracking change over time if both core and peripheral sites are included [84], which for the focal taxa of this study likely would entail sites distributed across an elevational gradient [85]. In addition, for taxa like *Plethodon*, sampling designed to monitor changing patterns of hybridization would also be informative [68,86]. All things considered, genomic capture panels such as the one we present appear to represent a viable tool for monitoring salamanders [25].

## Supporting information

S1 File Tables S1 and S2, description of tissue sampling protocol, data and R code for all analyses, results from STRUCTURE harvester. (ZIP)
