# Supplementary material for: Development of genomic markers for monitoring and research on plethodontid salamanders
Source: PLoS One. 2025 Nov 6;20(11):e0336236. doi: 10.1371/journal.pone.0336236 (PMC12591486; doi:10.1371/journal.pone.0336236)
Supplement: S1 File — (ZIP) [file pone.0336236.s001.zip › S1_file/Tables S1 and S2.docx]

Supplemental tables:

Development of genomic markers for monitoring and research on plethodontid salamanders

Benjamin M. Fitzpatrick^1^*, Kara S. Jones^2^, Aaron W. Aunins^2^, Michael S. Eackles^2^, David C. Kazyak^2^

^1^ Department of Ecology and Evolutionary Biology, University of Tennessee, Knoxville, TN, 37996, USA

^2^ U.S. Geological Survey, Eastern Ecological Science Center, Leetown Research Laboratory, 11649 Leetown Road, Kearneysville, WV, 25430, USA

* Corresponding author

Email: benfitz@utk.edu

Disclaimer: Any use of trade, firm, or product names is for descriptive purposes only and does not imply endorsement by the U.S. Government.

Table S1. Source of salamander DNA samples used in the two capture kit tests.

| **Sample** | **Species** | **Latitude** | **Longitude** | |
| --- | --- | --- | --- | --- |
| **First test** | | | | |
| 39B | *Desgmognathus aeneus* | 34.79 | -83.93 | |
| 18B | *D. folkertsi* | 34.83 | -83.73 | |
| HBS03 | *D. monticola* | Not recorded | | |
| HBS05 | *D. ocoee* | 35.09 | -83.82 | |
| 34B | *D. quadramaculatus* (northern) | 37.71 | -81.53 | |
| 14B | *D. quadramaculatus* (southern) | 34.80 | -84.60 | |
| HBS06 | *D. wrighti* | 36.23 | -81.51 | |
| HBS13 | *Eurycea guttolineata* | 35.3 | -82.77 | |
| HBS18 | *Gyrinophilus porphyriticus* | 35.05 | -83.19 | |
| PM316 | *Plethodon montanus* | Not recorded | | |
| **Second test** | | | | |
| GSM19044 | *Plethodon metcalfi* | 35.631 | -83.046 | |
| GSM19095 | *P. metcalfi* | 35.572 | -83.162 | |
| GSM19101 | *P. teyahalee* | 35.585 | -83.119 | |
| GSM19113 | *P. glutinosus* | 35.773 | -83.180 | |
| GSM19401 | *P. teyahalee* | 35.527 | -83.922 | |
| GSM19407 | *P. glutinosus* | 35.610 | -83.806 | |
| PJO119 | *P. jordani* | 35.550 | -83.495 | |
| PJO291 | *P. jordani* | 35.570 | -83.679 | |
| PJOIK7 | *P. jordani* | 35.727 | -83.238 | |
| PJOYCG3 | *P. jordani* | 35.725 | -83.242 | |
| DWR12 | *D. wrighti* | 35.542 | | -83.494 |
| DWR163 | *D. wrighti* | 35.526 | -83.855 | |
| DWR63 | *D. wrighti* | 35.697 | | -83.126 |

Table S2. Results from the first capture kit test showing the total number of reads that were sequences, the number and percentage of those reads that were primary mapped, and the final number of percentages of reads that were mapped after quality control measures to remove reads that were duplicates, not properly paired, or where mates mapped to different chromosomes. Final mapped reads indicates how many sequenced reads were on target following the capture process.

|  | **Total reads before mapping** | **Primary mapped reads** | | **Final mapped reads** | |
| --- | --- | --- | --- | --- | --- |
| *D. aeneus* | 41,563,469 | 20,481,129 | 49% | 7,307,696 | 18% |
| *D. folkertsi* | 95,445,419 | 48,879,130 | 51% | 15,582,579 | 16% |
| *D. monticola* | 32,924,928 | 16,611,083 | 50% | 7,082,936 | 22% |
| *D. ocoee* | 35,645,627 | 19,243,064 | 54% | 7,642,794 | 21% |
| *D. quadramaculatus* (northern) | 100,081,552 | 52,398,382 | 52% | 16,414,037 | 16% |
| *D. quadramaculatus* (southern) | 27,540,456 | 13,047,859 | 47% | 5,625,570 | 20% |
| *D. wrighti* | 74,173,047 | 34,925,495 | 47% | 11,444,766 | 15% |
| *E. guttolineata* | 68,351,828 | 6,770,718 | 10% | 2,394,757 | 4% |
| *G. porphyriticus* | 71,023,400 | 11,808,850 | 17% | 3,607,017 | 5% |
| *P. montanus* | 54,446,103 | 11,258,424 | 21% | 3,920,837 | 7% |
