# Supplementary material for: Development of genomic markers for monitoring and research on plethodontid salamanders
Source: PLoS One. 2025 Nov 6;20(11):e0336236. doi: 10.1371/journal.pone.0336236 (PMC12591486; doi:10.1371/journal.pone.0336236)
Supplement: S1 File — (ZIP) [file pone.0336236.s001.zip › S1_file/Sampling_Methods_Appendix.docx]

Supplemental Methods

**Development of genomic markers for monitoring and research on plethodontid salamanders**

Benjamin M. Fitzpatrick^1^*, Kara S. Jones^2^, Aaron W. Aunins^2^, Michael S. Eackles^2^, David C. Kazyak^2^

^1^ Department of Ecology and Evolutionary Biology, University of Tennessee, Knoxville, TN, 37996, USA

^2^ U.S. Geological Survey, Eastern Ecological Science Center, 11649 Leetown Road, Kearneysville, WV, 25430, USA

* Corresponding author

Email: benfitz@utk.edu

**Justification for sampling protocols and adherence to legal and ethical standards**

Tissue sampling was carried out in compliance with the United States Animal Welfare Act [7 U.S.C. 2131 et seq.] and according to University of Tennessee Institutional Animal Care and Use Committee (IACUC) protocol 2710 and National Park Scientific Research Collection Permits GRSM-2019-SCI-2079 and GRSM-2019-2081.

Prior to this study, tissue samples were not available for the range-wide sampling required by our study design, therefore we could not replace live animal sampling with alternative study methods. Therefore, we minimized harm to animals by using the clean pinch method of tissue collection, which removes a small amount of tissue from live animals in the field and allows them to be released immediately at the point of capture (Shaw et al. 2025). The clean pinch method is applied with the animal in a clean zipper-lock bag to avoid skin damage and water loss. We apply a firm, gradual pinch through the bag to the distal part of the tail (0.5 to 1.0 cm) with fingers or a smooth-edged instrument until the animal autotomizes the tail tip. This is done rapidly (under 1s) and without tearing the bag. Once the salamander has autotomized the tail tip, the animal released alive as quickly possible, and the tissue sample is transferred to a labeled sample vial for transport.

Rainey et al. (2024) compared toe clips, buccal swabs, and skin swabs as DNA samples for a small species of frog. They found that DNA yield and quality were significantly better with toe clips, and that skin swabs were particularly poor sources of animal DNA. For genome scale DNA technologies, large amounts of high-purity DNA are critical to the success of the laboratory methods, and poor samples are often wasted (meaning the animal was handled for no benefit whatsoever). Moreover, buccal swabs require significantly more coercive handling than tail samples, with non-trivial risk of damage to the teeth and tongue, which are critical for effective feeding.

For salamanders, tail samples obtained by the clean pinch method are far less damaging to the animals than toe clips but provide the same sample quality. Polich et al. (2013) and Segev et al. (2015) found no evidence that tail-clipping affected growth and survival of larval salamanders, which is noteworthy considering the importance of the tail in swimming. Adult salamanders autotomize sections of their tails in response to localized pinching by physically releasing the nearest proximal myoseptum (Dinsmore 1977). When autotomy occurs on these natural cleavage planes, there is minimal bleeding, and the wound is rapidly sealed by intrinsic physiological mechanisms. Dinsmore (1977) found that regeneration after natural autotomy was faster and less likely to scar muscle tissue than after surgical tail clipping (e.g., with scalpel or scissors).

**References**

Beaupre SJ, Jacobson ER, Lillywhite HB, Zamudio K. 2004. Guidelines for Use of Live Amphibians and Reptiles in Field and Laboratory Research, 2nd ed. American Society of Ichthyologists and Herpetologists.

Dinsmore CE. 1977. Tail regeneration in the plethodontid salamander, Plethodon cinereus: induced autotomy versus surgical amputation. *J Exp Zool*. 199:163–75. DOI: 10.1002/jez.1401990202.

Polich RL, Searcy CA, Shaffer HB. 2013. Effects of tail-clipping on survivorship and growth of larval salamanders. *J Wildl Manage* 77:1420–1425. DOI: 10.1002/jwmg.596

Rainey TA, Tryc EE, Nicholson KE. 2024. Comparing skin swabs, buccal swabs, and toe clips for amphibian genetic sampling, a case study with a small anuran (*Acris blanchardi*). *Biol Methods Protoc*. 16;9:bpae030. DOI: 10.1093/biomethods/bpae030.

Segev O, Polevikove A, Blank L,Goedbloed D, Küpfer E, Gershberg A, et al. 2015. Effects of Tail Clipping on Larval Performance and Tail Regeneration Rates in the Near Eastern Fire Salamander, *Salamandra infraimmaculata*. *PLoSONE* 10: e0128077. DOI:10.1371/journal.pone.0128077

Shaw, A., R. Chastain, and B. M. Fitzpatrick. 2025. Geographic distributions and patterns of co-occurrence among black-bellied and shovel-nosed salamanders (Desmognathus spp.) in the Great Smoky Mountains National Park. PeerJ 13.
